# Understanding internet-supported self-management for low back pain in primary care: a qualitative process evaluation of the SupportBack 2 randomised controlled trial

**DOI:** 10.1136/bmjopen-2025-103428

**Published:** 2025-10-20

**Authors:** Adam W A Geraghty, Stephanie Hughes, Lisa Roberts, Jonathan C Hill, Nadine E Foster, Elaine Hay, Gemma Mansell, Malcolm White, Firoza Davies, Mary Steele, Paul Little, Lucy Yardley

**Affiliations:** 1 Primary Care, Population Sciences and Medical Education, University of Southampton, Southampton, UK; 2 School of Health Sciences, University of Southampton, Southampton, England, UK; 3 Primary Care Centre Versus Arthritis, School of Medicine, Keele University, Newcastle-under-Lyme, England, UK; 4 STARS Education and Research Alliance, The University of Queensland and Metro North Health, Brisbane, Queensland, Australia; 5 School of Health & Life Sciences, Aston University, Birmingham, UK; 6 PPI, University of Southampton, Southampton, England, UK; 7 School of Psychology, University of Southampton, Southampton, England, UK; 8 School of Psychological Science, University of Bristol, Bristol, England, UK

**Keywords:** Internet, Self-Management, Digital Technology, Back pain, QUALITATIVE RESEARCH

## Abstract

**Abstract:**

**Objective:**

The SupportBack 2 randomised controlled trial (RCT) compared the clinical and cost-effectiveness of an internet intervention supporting self-management versus usual primary care in reducing low back pain (LBP)-related disability. In this study, we aimed to identify and understand key processes and potential mechanisms underlying the impact of the intervention.

**Design:**

This was a nested qualitative process evaluation of the SupportBack 2 RCT (ISRCTN: 14736486 pre-results).

**Setting:**

Primary care in the UK (England).

**Participants:**

46 trial participants experiencing LBP without indicators of serious spinal pathologies (eg, fractures, infection) took part in telephone interviews at either 3 (n=15), 6 (n=14) or 12 months (n=17) post randomisation. Five physiotherapists who provided telephone support for the internet intervention also took part in telephone interviews.

**Intervention:**

An internet intervention ‘SupportBack’ supporting self-management of LBP primarily through physical activity and exercise delivered in addition to usual care, with and without physiotherapist telephone support.

**Analysis:**

Data were analysed thematically, applying a realist logic to develop context-mechanism-outcome configurations.

**Results:**

Four explanatory themes were developed, with five context-mechanism-outcome configurations. Where benefit was reported, SupportBack appeared to work by facilitating a central associative process where participants linked increases in physical activity or exercise with improvements in LBP, then continued to use physical activity or exercise as key regulatory strategies. Participants who reported little or no benefit from the intervention appeared to experience several barriers to this associative process, including negative expectations, prohibitive beliefs about the cause of LBP or functional limitations preventing engagement. Physiotherapists appeared to provide accountability and validation for some; however, the remote telephone support that lacked physical assessment was viewed as limiting its potential value.

**Conclusions:**

Digital interventions targeting physical activity and exercise to support LBP self-management may rely on mechanisms that are easily inhibited in complex, heterogeneous populations. Future research should focus on identifying and removing barriers that may limit the effectiveness of digital self-management support for LBP.

**STRENGTHS AND LIMITATIONS OF THIS STUDY**

- This qualitative process evaluation was nested within the largest trial to date of an internet intervention designed to support self-management of low back pain in primary care.
- The qualitative analysis was designed to be explanatory, supporting application of the findings beyond the trial.
- The findings are based on the experiences of participants who completed trial processes; perceptions and experiences may differ in those who dropped out of the trial early.

## Introduction

 Low back pain (LBP) remains the leading cause of disability globally.^1^ International guidelines have moved away from recommending medical and surgical management of LBP, with behavioural self-management now a key first-line care strategy.^2 3^ Self-management for LBP often comprises pain education, reassurance and support for increasing physical activity.^2^ While physiotherapists have a central role in supporting self-management for people experiencing LBP,^4^ the global scale and impact of the condition has led to increased research on potentially scalable digital self-management support.

Systematic reviews of digital LBP self-management randomised controlled trials (RCTs), including mobile apps and internet interventions delivered through websites, have shown mixed results. Review authors conclude that studies often had methodological issues, including use of many differing outcomes.^5 6^ Nicholl *et al*. stressed the need for large scale trials using core outcome sets and comparing the interventions versus usual care rather than no-treatment/waiting list controls^6^ to allow more robust conclusions on the effectiveness of digital self-management support for LBP.

Two large research programmes, selfBACK^7^ and SupportBack,^8^ had aims to determine the effectiveness of differing digital approaches for LBP self-management compared with usual care through large-scale RCTs. The selfBACK group’s intervention was a mobile app featuring machine learning with an activity monitoring wristband.^9^ Our group’s intervention (SupportBack) was internet-based and delivered through a website accessible through any device with access to the internet.^10^ Both interventions centred on general physical activity and specific exercises as the primary self-management strategies supported digitally. We additionally investigated remote physiotherapist support alongside our internet intervention. In both RCTs, the average between-group effects of the interventions on LBP-related disability on the primary outcome (Roland Morris Disability Questionnaire (RMDQ)^11^) were small, and did not reach prespecified minimal clinically important differences (MCID) compared with usual care (<1.0 in each case where Minimum Clinically Important Difference (MCIDs) ranged from 1.5 to 2.0^7 8^). Additionally, the remote physiotherapist support in the SupportBack 2 trial did not lead to greater benefit than when the internet intervention was delivered without this support. In both trials, there were indications of some benefit, that is, small but statistically significant improvements in LBP-related disability at 3 months in the selfBACK trial^12^ and cost-effectiveness in the SupportBack trial (particularly for the unsupported intervention).^13^ The average between-group effect of these digital interventions on LBP-related disability compared with usual care was very modest.

It is important to understand the processes that may have been responsible for the limited average benefit of these digital approaches to supporting self-management for LBP. The WHO and the European Union continue to suggest that digital health solutions are a critical complement to traditional services for chronic conditions,^14^ and funding continues to be awarded for digital musculoskeletal services in the UK^15^ and elsewhere. In this paper, we present the qualitative process evaluation of the SupportBack 2 trial. We aimed to understand the processes underlying outcomes, drawing from the experiences of participants and physiotherapists involved in the trial.

## Method

### Design

We present a nested qualitative process study as part of the SupportBack 2 RCT (ISRCTN14736486).^13^ We followed the Standards for Reporting Qualitative Research.^16^ The SupportBack 2 RCT was designed to determine the clinical and cost-effectiveness of an internet intervention in supporting self-management of LBP versus usual primary care. The trial had three arms: (1) usual care, (2) usual care plus the SupportBack intervention and (3) usual care plus SupportBack plus telephone physiotherapist support. The primary outcome was LBP-related disability measured by the RMDQ over 12 months. To be eligible, participants had to have consulted primary care with LBP; have current LBP at the time of recruitment without indicators of serious spinal pathology (such as fracture, infection, cauda equina); have access to the internet; and be able to read/understand English. Those who had spinal surgery within 6 months of recruitment or who were pregnant were also excluded.^8^

The internet intervention ‘SupportBack’ has been described in full elsewhere.^10 17^ Briefly, the aim of the SupportBack intervention was to support self-management of LBP through general physical activity with a specific focus on walking and back exercises. The intervention was based primarily around self-efficacy and self-regulatory theory.^18 19^ Cognitive reassurance, graded goal setting, self-monitoring and tailored feedback were used to support increases in walking or back exercises (or both). Each week, participants could set goals, receive automated feedback and adjust or continue with their goals. Primarily, self-tailoring was encouraged, with participants selecting activities and goals based on their preference, amidst some simple automated tailoring, for example, goal ranges differed at two levels depending on how limiting participants reported their LBP was that week. At the end of each week, they could select an additional module providing advice and supporting behaviour change on a particular LBP-related topic (including relieving pain; flare-ups; work; sleep; mood; daily living). The intervention comprised six online sessions designed to be delivered once per week. The primary content regarding physical activity was weighted to the first one-to-two sessions, such that sessions that followed for the most part built on and reinforced earlier messages. After the six sessions, the intervention became a static repository with the physical activity/exercise and educational modules that participants could access for the remainder of the trial.

In addition, those in the telephone-supported arm received up to three calls with a physiotherapist (up to an hour in total: one initial call of up to 30 min and two follow-ups of up to 15 min each). The calls were designed to be delivered over the 6-week primary intervention period. Physiotherapists were asked to adhere to a protocol focused primarily on providing reassurance, supporting engagement with SupportBack related goals and addressing concerns. They were asked not to provide an individualised assessment of the LBP presentation.

Within this process evaluation, trial participants were interviewed at three time points: after 3 months, 6 months and 12 months post randomisation. Different participants were interviewed at each point. This enabled us to qualitatively explore processes and the impact of the intervention at different durations from the primary interactive 6-week intervention period.

### Sampling and recruitment

Trial participants: We purposively sampled trial participants who had consented to take part in interviews as part of the trial procedures. We aimed for diversity in age, gender, symptom severity (physical function and pain duration) and high/low usage of the internet intervention. Analysis began in parallel to the interviews to ensure a diverse and broad range of responses from participants. The concept of information power^20^ was then used to inform when recruitment at each time point was deemed sufficient to meet the aims of the process evaluation. According to the information power model, sufficiency in a qualitative study with a ‘narrow’ study aim and a high level of sample specificity (ie, a process evaluation within an RCT) is likely to be reached with relatively modest numbers. Within this context, ‘quality of dialogue’ was a key item used in deciding when to stop recruiting.

Physiotherapists: 12 physiotherapists were involved in providing telephone support over the course of the trial, and all physiotherapists who were contactable were invited for an interview after the intervention period for trial participants (attempting total population sampling). Those physiotherapists who responded provided online consent ahead of their interviews.

### Interviews

Telephone interviews were conducted for both the trial participants and the physiotherapists by experienced qualitative researchers SH and MS, who had no prior relationship with the participants. The interviews were semi-structured, drawing on topic guides that were developed by the full team with input from the public contributors (see online supplemental file 1 for the topic guides). For participants, the interviews initially covered history of LBP and previous treatment experiences, before moving to the central part of the interview, focusing on participants’ experiences of the SupportBack intervention (including digital aspects of SupportBack as well as implementing the activity suggestions), telephone support and usual care. The interview topic guides were very similar for the 3-month and 6-month interviews. We added new questions regarding longer-term implementation of activities for the 12-month interviews for those in the intervention arms. For physiotherapists, interviews focused on their experiences of delivering the support, their perspectives on the general approach (remotely supporting digital self-management) and any issues or barriers. Interviews were audio recorded and transcribed verbatim.

### Analysis

We conducted a thematic analysis applying a realist logic.^21 22^ Initially, analysis began inductively with an independent double coding of the participant interviews by SH and AWAG, with SH coding the physiotherapist interviews (using NVivo software). The initial coding was discussed and agreed with the wider team (LY, LR, PL, JCH and public contributors FD and MW) using codebooks with extensive data examples. This was conducted prior to the main trial outcomes being known. For the present analysis, the aim was to develop these codes into broad explanatory themes drawing on realist logic, for example, working to explain the apparent effects of the SupportBack intervention in the trial. Abductive reasoning (seeking the ‘best explanation’ for the data) and retroductive reasoning (inferring possible deeper ‘hidden’ mechanisms and their associated contexts)^23 24^ were used to develop themes and associated ‘context-mechanism-outcome’ (CMO) configurations. CMOs are relatively brief statements used in realist analysis to summarise aspects of explanatory, intervention-specific theory regarding the effect of an intervention.^25^ Data were analysed sequentially; draft explanatory themes for the trial participant data were developed first. These draft themes were then ‘tested’ and expanded using the physiotherapist data. Explanatory themes including CMOs were initially drafted by AWAG and agreed with SH working closely with public contributors FD and MW. Broader input was then sought from LY, LR, PL, JCH, NEF, GM and EH to discuss, amend and confirm themes and CMOs. Despite the different interview time points for trial participants, their responses remained very similar at each time point. As a result, they were analysed together. Input into the analysis regarding longer-term impacts and processes was drawn primarily from the 6-month and 12-month interviews.

### Patient and public involvement

Public contributors MW and FD both have lived experience of LBP. They were involved in the delivery and interpretation of the RCT findings as well as the qualitative process analysis. Prior to MW and FD joining the team, we had a group of three different public contributors with lived experience of LBP who co-developed the interview topic guides as part of our process team. MW and FD were involved in all qualitative process evaluation meetings once data collection began and discussed and agreed early coding manuals alongside descriptive aspects of the analysis. MW and FD contributed to both the development of the overarching programme theory and the development of the specific CMOs that follow. They are coauthors and contributed to and agreed the current manuscript.

### Reflexivity

A combined/integrated approach^26^ to the process and outcome evaluation meant that the same team delivered both the trial outcomes evaluation and the process evaluation. Our team is a mix of academic psychologists and physiotherapists, an academic general practitioner and rheumatologist alongside public contributors with lived experience of LBP. The team has been in equipoise about the potential impact of internet-based self-management and SupportBack is not a commercial product. The full team with a range of differing perspectives agreed the final analysis and interpretations presented here.

### Trustworthiness

Many of the above approaches were used to enhance the trustworthiness^27^ of the analysis: Initial double coding; sharing of detailed codebooks across the team including public contributors; triangulation of the ongoing analysis with public contributors and multidisciplinary coauthors; sequential analysis of trial participant and physiotherapist participant data, and a reflexive approach throughout.

## Results

92 trial participants were invited to interview (from the total RCT sample, n=825) across three time points within the trial (3 months, 6 months and 12 months post randomisation). 46 participants agreed to interview (3 months n=15, 6 months n=14 and 12 months n=17). Interviews ranged in duration from 19 to 59 min, and qualitative data were collected between March 2020 and November 2021 (quotes are provided with details of ID, interview time point, baseline LBP episode duration, and trial arm). Of the 12 trial physiotherapists contacted, 4 had changed email addresses and 3 did not respond. The remaining five trial physiotherapists were contactable and agreed to be interviewed (42%). Interview duration ranged from 18 to 41 min, and physiotherapist data were collected between September and October 2021. See table 1 for characteristics.

**Table 1:** Trial participant and physiotherapist characteristics

Trial participant characteristics	N/M	%/SD
Gender, n (%)		
Male Female	26 20	56.5 43.5
Age, mean (SD)	56.5	13.7
Education, n (%)		
No response No formal qualifications GCSE/O levels or similar A levels or similar or ONC/OND HNC/HND degree Degree Higher degree Postgraduate degree	1 2 9 5 8 9 3 8	2.2 4.4 19.6 10.9 17.4 20.0 6.5 17.4
Other	1	2.2
Ethnicity, n (%)		
White	39	92.9
Black	3	7.1
Missing	4	8.7
Baseline RMDQ, mean (SD)	6.5	5.5
Pain duration, n (%)		
Less than 3 months 3–6 months 7–12 months 1–2 years 3–5 years 6–10 years Over 10 years	9 4 8 5 11 3 6	19.6 8.7 17.4 10.9 23.9 6.5 13.0
Physiotherapist characteristics		
Gender		
Male Female	1 4	20 80
Age	41.2	10.0
Band*		
6 7 8a	1 3 1	20 60 20
Years qualified	19.6	9.61
Mean years working in musculoskeletal services	14.4	5.94

* In the UK, health professionals are graded according to their theoretical knowledge and clinical experience. This system has ‘bands’ from 1 to 9 and is applied to clinical and support roles (higher numbers reflecting greater experience).

GCSE, General Certificate of Secondary Education; HNC, Higher National Certificate; HND, Higher National Diploma; O, Ordinary Level; ONC, Ordinary National Certificate; OND, Ordinary National Diploma; RMDQ, Roland Morris Disability Questionnaire.

### Section 1: understanding the SupportBack intervention

Generally, participants reported finding the SupportBack intervention website easy to use, with a clear and simple layout. Although digital sessions were recommended to be used once per week for 6 weeks, participants reported engaging with the sessions in different ways that suited them. Some reported using the sessions weekly, as designed, some ‘dipped in and out’ and others used early sessions to write down what they found useful and did not return to the website. It was common for participants to describe the usefulness of physical activity explanations and clear videos, although some reported that they would have preferred greater tailoring and individualisation. While some participants described their use of the LBP-related modules (eg, mood, sleep), discussion of these modules was infrequent.

#### Explanatory theme 1: supporting a positive experiential learning process

There was no consistent pattern reported in prior LBP severity, functional limitations or pain duration in participants describing benefit following the SupportBack intervention. This aligned with the quantitative findings, where baseline risk of persistent disability and pain duration did not modify outcomes.^13^ Participants describing benefit did, however, report an apparent openness to the intervention; ‘I honestly didn’t know what to expect. I just thought, give it a try—if you don’t try it, you’ll never know’ (S0952). This receptiveness seemed to be related to limited previous experience of physiotherapy and was also linked to prior experience of physical activity including exercise being helpful. For these participants, use of SupportBack appeared to support growing confidence and reassurance in the use of physical activity or exercise as a primary management strategy for their LBP. This was facilitated through trusted suggestions within the material, and more specific guidance to help with adjustments to back exercises. Some participants also reported that prompting via automated emails, or forthcoming phone calls for those in the supported arm, increased motivation to engage in physical activity/exercise.

I: What do you think was the most useful aspect of the SupportBack website?

P: I think it gave me the confidence to try different exercises, and know that they come from somebody that knows what they’re talking about, rather than perhaps looking up on a website on your tablet or computer. Saying, ‘Oh, that one looks quite good,’ but it has come from an unknown source somewhere, and somebody said it works. If it came from a genuine source was absolutely essential.

S0979 12 months, episode duration <3 months (telephone support)

So but, in terms of the support programme, what I liked that was good about that was there was an expectation that you would do it. I wasn’t forced to do it, but there was like, you’re on the programme, this is what you should be doing and having somebody —in my case—phone me up is good to have some external incentive, rather than just your own incentive to do things.

K0504 3 months, episode duration 3–5 years, (telephone support)

Telephone physiotherapist support was often reported as contributing to these initial processes including reassurance, confidence and motivation:

As I said she was really easy to talk to. Very approachable with any questions I had for her. Very encouraging to say that yes, you are doing all the right things, keep it up.

K0693 6 months, episode duration 1–2 years (telephone support)

The telephone support physiotherapists stressed that they felt the accountability of the forthcoming calls for those in the supported arm was central in participants engaging with physical activity or exercise.

They liked having the phone call contact, as I say, both during it and I think then as a motivator towards then carrying on and completing the programme. In that they felt supported that someone was going to be checking in on them.

Physiotherapist ID08

Increased confidence, reassurance and motivation appeared to support physical activity/exercise attempts as part of the intervention. The perception that these attempts positively influenced LBP seemed to constitute a central mechanism leading to reported benefits: The explicit linking of increases in physical activity/exercise with the experience of reduced pain or improved mobility. This learning mechanism, including the experience of LBP returning when activity stopped, appeared to be key in reports of sustained engagement with physical activity/exercise. This learnt process seemed to become part of a regulatory strategy that resulted in reports of long-term improved function and reduced pain.

I do part of those exercises which I’ve found very good because I hadn’t realised that those exercises could coordinate with the relief of the pain in my back. They did the job, the more I learnt, the more I did your exercises, the more it become a lot easier for me and my back.

S1116 6 months, episode duration 3–5 years (website alone)

Well, I did some exercises there; I think I put down the ones that I was very happy with, and I kept them up but as soon as I stopped, it started to come back again. So if I forget and my back starts hurting, I think to myself, oh, I haven’t done my exercises! So I spend ten minutes, quarter-of-an-hour doing my exercises.

S1197 12 months, episode duration 3–6 months (website alone)

Int: Do you still do the SupportBack exercises?

P: Yes.

Int: Okay, that’s great. What motivates you to keep doing them?

P: The fact that I’m not having the amount of pain that I used to get in my back. [The exercise] has strengthened up my back muscles and I aim to keep them as strong as possible, to keep me mobile and out of pain.

S0979 12 months, episode duration <3 months (telephone support)

Within this sample, there seemed to be a preference for trying the back-specific exercises over walking. Some participants suggested that the lack of novelty of walking steered them towards back exercises, others suggested that walking for long periods increased their LBP. Nevertheless, those that did favour walking or used walking alongside exercises described a similar linking of walking activity with improvements and use of walking as a regulatory strategy for LBP.

I preferred the walking. The back exercises I felt were a necessary thing to help me get better […] I think the back exercises maybe helped a little bit more. I don’t know, they both helped. I certainly felt better when I went walking back-wise most of the time.

S0914 6 months, episode duration 1–2 years (telephone support)

Int: Did you prefer the back exercises or the walking?

P: Walking. Well, I like walking anyway, and I do quite a lot of it, easy to fit in if you like. Yes, so before the trial, if I had a flare-up, I would have taken painkillers until it stopped hurting. I wouldn’t have taken to my bed, but I certainly would have gone and sat down and tried to rest it. Now I try to manage it with paracetamol and going for a walk and, I’m touching wood now, I haven’t had a bad back flare-up since before the trial.

K0787 12 months, episode duration: over 10 years (website alone)

Participants in the supported arm described how the physiotherapists could tailor and personalise the activity/exercise suggestions on the website. Physiotherapists described how they could help participants with their own problem solving and work around problems. For some, this tailoring and problem solving may have further increased the likelihood of perceiving an association between physical activity/exercise and improvement; resolving issues and steering participants away from activities they deemed less likely to help.

I think he was just really understanding, and he explained some of the exercises that I would find better for my back, rather than some of the other exercises. When I said I found some didn’t really do anything and they gave me backache, he explained about the pressure on it and that, so he was quite helpful explaining to me about which exercises would probably be better for me.

S0650 3 months, episode duration <3 months (telephone support)

They like relaying details about it, and then you can come up with a strategy. So if they did find it difficult, sometimes, it’s then just articulating how they found it difficult. They probably know the answer, but it’s the whole articulating process. Then they can think, okay, well, maybe I should walk a different route, or maybe I should do—they come up with their own strategy.

Physiotherapist ID05

**CMO 1:** A psychological receptivity and openness to trying SupportBack (context), coupled with use of the SupportBack intervention (resource) appeared to increase confidence, provide reassurance and motivation for physical activity and exercise as support for LBP (mechanism). These processes, which were also facilitated by the physiotherapist in the supported arm, enabled the experiential association of increased physical activity or exercise with reductions in LBP and related limitations (mechanism). This learnt association appeared to lead to longer-term engagement with physical activity/exercise as a regulatory process and descriptions of general improvements in LBP (outcomes).

#### Explanatory theme 2: unable to impact LBP through activity

Where participants reported limited or little benefit from the SupportBack intervention, there appeared to be common prior experiences affecting intervention processes. Some participants described long histories of engaging with physiotherapy with limited or mixed success. Generally, these participants reported that physical activity or exercise had limited impact on their LBP. These perceptions seemed to be driven by previous unsuccessful attempts with activity-based approaches, including physiotherapy and yoga. Some described that their LBP was gradually worsening and/or that they would always have it. This expectation of ongoing chronicity was linked to beliefs about the physical cause of LBP (eg, degeneration of the spine) in some cases.

I: What’s been the least helpful treatments that you’ve tried?

P: To be perfectly honest, physio. I know it sounds counterintuitive, but I’ve been doing stretching exercises for, since I’ve had this issue. It’s one of those. I’m still doing them to this day, but my pain, it obviously increases and decreases depending on what the situation is. These stretches and exercises I haven’t felt have made a massive difference.

S1111 6 months, episode duration 6–10 years (telephone support)

As I say, I think it’s—shall we say, [SupportBack]—I don’t think it was ever going to cure the problem, because as I say, it’s degradation of the vertebrae, so there’s no way I’m going to rebuild the vertebrae in the right shape they ought to be.

S1018 6 months, episode duration 7–12 months (telephone support)

Physiotherapists also described participants’ perceived severity to be a limiting factor, acting as a barrier to engagement with the activities:

I suppose the ones that didn’t seem to engage with it were the ones that had more severe problems, I would say; and they themselves said, ‘I’m not sure this is appropriate for me, because I can’t do a lot of this.’ Yes, I think it probably would be the ones that we’re more severe.

Physiotherapist ID04

Participants’ complex histories with managing their LBP influenced their expectations of the SupportBack intervention. For some, negative expectations limited their engagement with the physical activity/exercise suggestions within the programme. For others, they did engage, trying activities although with low expectations of benefit. Some participants’ low expectations appeared to be compounded by allocation to SupportBack without physiotherapist support.

When I got put in the randomised group of not having the phone consultations, I was like, oh—I then did the first week, and then I was—this isn’t going to be a game changer is what I thought.

S0882 3 months, episode duration 3–5 years (website alone)

Although there was some variability in the reports of those that did not describe overall benefit from SupportBack (eg, some aspects were reported as helpful such as reminders and clear videos of exercises), generally, many of the narratives featured a lack of an experiential linking between physical activity/exercise and improvements in LBP. This was evident in past reports of physical activity/exercise attempts, or in a lack of reported benefit when trying the activities in SupportBack. Despite this, some carried on with physical activity or exercise suggestions, fearing worsening of pain if they stopped. Other participants appeared to suggest a disengagement from activity attempts, moving to a process of tolerating LBP.

Normally I do gentle stretching exercises and things like that. I don’t go overboard with this, for the simple reason I found if I start going overboard, it gets worse. That’s the reason why over the last, say, two years it’s progressively got worse now to a stage where no matter, even if do the exercise, it doesn’t make a blind bit of difference.

K0704 12 months, episode duration <3 months (website alone)

Like I say, I put up with it now so I’m not really exploring it anymore, I’m just putting up with it now. I’m not looking to go on the website for anything more or asking for any more support.

S1241 12 months, episode duration 3–5 years (telephone support)

Those in the supported arm who reported little benefit overall often described the limitations of the telephone support. They talked about how they felt in-person physical contact was necessary with the physiotherapist. As above, these participants had experienced mixed or limited benefits of physical activity on their LBP. It appeared that they perceived their cases to have a greater experiential complexity (eg, physical activity/exercise *not* working to improve LBP). This additional complexity thus needed to be matched by the detail and intensity of the physiotherapist contact (physical assessment), which was viewed as lacking in the SupportBack intervention.

As I’ve said before, I think although it’s all done with the best will in the world, any form of physiotherapy, quite frankly, is going to be very limited if it’s not involving physical contact, physical examination and testing.

S1018 6 months, episode duration 7–12 months (telephone support)

**CMO 2:** Previous experience of unsuccessful physiotherapy for LBP, physical activity-based approaches that did not lead to benefit, or acceptance of long-term pain reduced expectations (context) regarding the utility of SupportBack (resource). This led to either disengagement or continued physical activity/exercise attempts without impact on LBP. Both constituted a learning experience or reinforced prior expectations that physical activity or exercise did not (or could not) reduce LBP (mechanism). These processes appeared to be central in participants reporting a lack of overall benefit in LBP-related function in the longer term following the intervention (outcome).

### Section 2: delivering remote telephone support with the SupportBack website

Generally, there were no major barriers to providing the telephone support described by physiotherapists. There were some minor implementation issues such as difficulty in contacting some of the participants, or participants favouring call times that were difficult for the physiotherapists. The physiotherapists reported feeling well-guided by the study team and equipped to deliver the support following their training as part of the trial.

#### Explanatory theme 3: validating support and encouraging responsibility

The physiotherapists’ descriptions suggested that when they perceived their role to work well, the provision of accountability, reassurance and space to listen to patients’ concerns was central. While accountability and reassurance have been discussed as an aspect of motivation within explanatory theme 1, physiotherapists perceived being ‘heard’ by a musculoskeletal professional as validating for the patients.

Well, I think it was because, as I said, the patients were being listened to, and someone wanting to actually engage in their rehab, so they were actually interested in their rehab.

Physiotherapist ID05

You think, right, okay, because they felt really happy that a qualified professional was ringing them and spending enough time with them that they felt that they were actually gaining from it. They felt that they had a bit of time with somebody who knew what they were talking about.

Physiotherapist ID09

**CMO 3:** Physiotherapists provided accountability and time to hear patients’ stories and concerns (context). Physiotherapists perceived this as validating and reassuring for the patients (mechanism), which appeared to support engagement with the intervention process (outcome).

Within this context of listening and support, some physiotherapists perceived their more limited role in the patients’ care as acting as a support for the self-management process. With a role as a supportive guide for specific LBP-related behaviour change, they felt the responsibility was clearly with the patient to lead their own rehabilitation. They often contrasted this with their experience of standard physiotherapy practice.

It’s self-directed and goal driven, I think it’s very much you’re feeding into the patient’s own wishes. I think there’s no, because we haven’t assessed them or done anything like that with them, there’s no bias from our side. So I think in terms of getting patients onboard to think of their own treatment goals and things like that, I think it’s particularly worthwhile for that.

Physiotherapist ID08

It’s breaking down that relationship perception that I am there as the font of all knowledge, and you’re there to ask me questions and gain knowledge from me. It’s trying to establish it the other way round. Get them to take onus of their progress and their progression across the weeks.

Physiotherapist ID09

**CMO 4:** Physiotherapists were more distant from patients’ care; for example, no access to medical history or physical assessment and their role was focused on specific behavioural support (context). Some physiotherapists perceived this placed greater responsibility for behaviour change on the patient, reducing passivity (mechanism) and increasing engagement with goals and activities (outcome).

#### Explanatory theme 4: restriction in physiotherapist support

Within the trial, the telephone physiotherapist support was specifically designed to be brief and was developed within a ‘CARE’ framework^28^ (‘Congratulate on any progress’, ‘Address concerns’, ‘Reassure’ and ‘Encourage progress toward goals’) for clinician support for digital interventions. Consequently, physiotherapists were not asked to provide individualised LBP assessment or recommend exercises/approaches beyond what was offered in the online SupportBack intervention. Some support physiotherapists who worked on the trial described difficulties that appeared to come from perceived distance from the patient, both physically and in terms of their understanding of individual patient’s clinical histories and previous management relating to LBP.

I think I would prefer to see them face-to-face to establish that relationship, and I think that would have helped to engage. I would like to have been the clinician that examined them first of all, assessed them, because that was slightly tricky, I think, because they had their different management beforehand that they wanted to talk about. I’ve got to say that lots of them did ask me about their management, and I know that wasn’t our role, but lots of them wanted to do that.

Physiotherapist ID05

Some physiotherapists described difficulties in keeping to their primarily supportive role within the trial, rather than offering care within their usual range of practice. They also perceived that some patients’ expectations for support were beyond what they had been asked to offer in the trial.

Some people were maybe under the impression that I was providing actual physio for them. That was the only negative, that sometimes people were asking me clinical questions about stretches of a certain limb, when we were really working on the online aspect and how you’re getting on with that. I think their expectations sometimes were a little bit, oh, now I’ve got hold of a physio, now I can ask all these questions!

Physiotherapist ID03

**CMO 5:** Within the trial protocol, physiotherapists were working remotely within a restricted scope, without access to patients’ histories and with a specific bounded focus for their advice/support (context). Physiotherapists perceived that some patients expected them to work beyond this role (mechanism) leading to potential dissatisfaction among patients and a frustration among physiotherapists (mechanism). For some, this misalignment and subsequent frustration appeared to limit therapeutic potential and may have impacted patient engagement (outcome).

**Figure 1:**
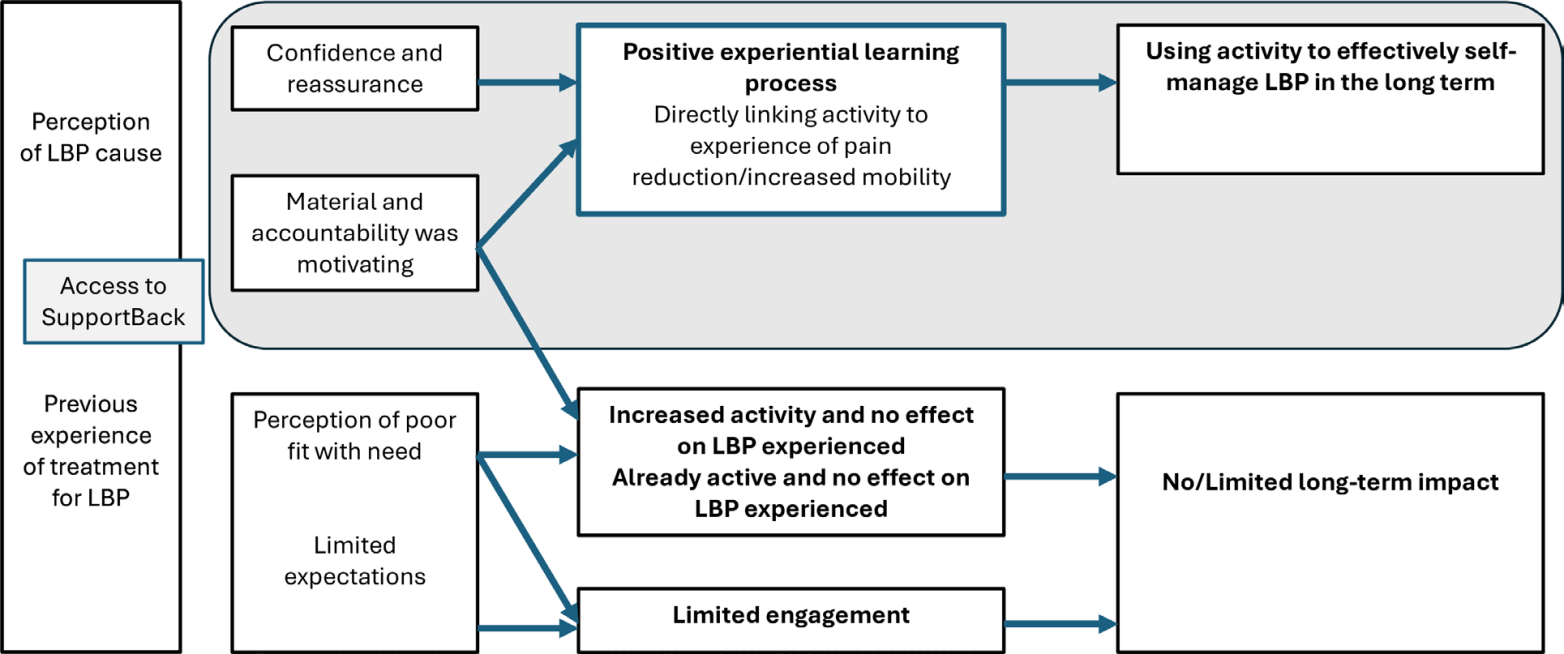
Programme theory representing central processes underlying impact (or lack of) from the SupportBack intervention. LBP, low back pain.

Figure 1 shows a representation of a programme theory focusing on the central processes from the CMOs relating to impact of the intervention on participants.

## Discussion

In this qualitative process evaluation, we aimed to understand the behavioural processes underlying the impact of the SupportBack intervention in the SupportBack 2 trial.^13^ Our analysis of the qualitative reports suggests a central associative and regulatory process: SupportBack appeared to provide a structured opportunity to associate physical activity and exercise with improvements in LBP or LBP-related functioning (akin to a behavioural experiment). Where this positive association was experienced, activity/exercise was then seemingly applied as a key regulatory strategy for LBP management moving forward. Although SupportBack offered more than physical activity and exercise guidance (eg, support for mood problems, sleep, LBP-related occupational issues), where benefit was discussed, it was this positive associative process that appeared central. Where limited benefit of SupportBack was described, participants talked through barriers that likely blocked this association between physical activity/exercise and LBP improvements; negative expectations, beliefs or functional limitations that would not allow for this apparent mechanism. Others engaged with suggested physical activity/exercise and experienced no benefits, with the lack of association leading to disengagement. The impact of physiotherapist support also appeared tied to this process. Physiotherapists provided extra accountability and reassurance to engage in physical activity or exercise, along with personalisation where participants experienced problems. However, trial participants and physiotherapists also discussed the limits of the telephone practitioner support approach, suggesting the need for and importance of face-to-face, ‘hands-on’ physical assessment and treatment for some patients.

More intensive physiotherapist-led interventions for LBP have shown greater reductions in LBP-related disability^29^ than in our SupporBack 2 trial. Such interventions target multiple psychophysiological mechanisms with a high degree of personalisation, in the context of a one-to-one relationship with a physiotherapist with extra training in these interventions.^30^ It seems digital support for self-management, focusing primarily on increasing physical activity or exercise, has inherently fewer mechanistic ‘levers to pull’ to achieve beneficial LBP outcomes. While remaining a theory based on qualitative data, the proposed central positive associative process^31^ underlying improvement following SupportBack is likely to be easily inhibited: Within a context of primarily online, automated support, there is little in place to manage inevitable challenges and complexities participants face when attempting to apply intervention suggestions. This is particularly the case in a large heterogeneous primary care sample with a wide range of LBP histories (including previous negative healthcare experiences) and social contextual sources of beliefs about spinal problems.

Although marginal, the quantitative data from the trial showed differences in the patterns of improvements in LBP-related disability over time in the telephone supported versus unsupported arms. In the supported arm, most benefit was reported around the time of the physiotherapist support, with little improvement after 3 months. Those in the unsupported arm continued to improve over time, with the greatest and statistically significant differences being seen over usual care alone at 12 months. Based on the CMOs and theory proposed here, one possible explanation for this is as follows: Physiotherapists talked of accountability being central to supporting participant motivation. It is possible providing then removing this external accountability after approximately 6 weeks unwittingly limited autonomous motivation. Additional telephone follow-ups may have extended improvements although at greater cost. For the unsupported arm, if this central associative process is key followed by the continued application of physical activity/exercise as a regulatory strategy for LBP, it is possible this would take time to develop and become habitual, aligning with observed gradual improvements.

A motivational systems approach^32^ may be helpful as a broad theoretical framework for understanding central behaviours in the trial. According to this framework, when attempting to self-manage pain, behaviour is guided by a complex dynamic control system.^32 33^ Control here refers to adjusting behaviours (‘behavioural outputs’) to bring experienced states closer to reference criteria or goals. A key aspect we have highlighted here is relatively simple, for example, people adjust behaviours and increase physical activity or exercise then monitor for improvements (pain reduction and/or increased mobility/function) to determine whether to continue with the behaviour or disengage. However, an important aspect of a motivational systems approach is that goals and control systems are multilevel and hierarchically organised,^34^ such that when to attempt a behaviour or when to disengage considering little improvement, will be guided by higher-order goals. These goals will be driven and influenced by a multitude of factors, factors which could be explored to remove barriers in clinical/interventional interactions. Critically, it is a dynamic, systems-based approach that can hold the acknowledged complexity that is part of managing LBP.^35^ Other behavioural theories or concepts have often been applied to LBP in relatively static ways such as self-efficacy theory^36^ or the mapping of behaviour change techniques.^37^ Application of more dynamic behavioural theory may support increasing effectiveness of self-management interventions.

The selfBACK team’s process evaluation focused primarily on barriers/facilitators to implementation and engagement with their app,^38^ rather than explanatory behavioural analysis. Nevertheless, there were several similarities: Svendsen *et al* report that participants who appraised the selfBACK app positively were those who favoured self-management, found the material motivating and reassuring, and reported positive effects on pain. Those in their study who appraised the app negatively and had limited engagement did not ‘buy into’ self-management as key, felt personalisation was limited, did not experience positive effects on their pain and wanted more interaction with a health professional.^38^ The parallels across the two programmes suggest similar underlying processes and need to focus on removing barriers to improve the overall effectiveness of digital self-management interventions for LBP.

There are several strengths of this work. The qualitative analysis draws on interviews with participants at different lengths of time from the primary interactive element of SupportBack, from 3 months to 12 months, providing the opportunity for a range of experiences of integration of strategies over the longer term. Our public contributors with lived experience of LBP were involved at all stages of the qualitative analysis, from reviewing codes to collaborating on CMO development. Mirroring the trial, we recruited participants with a diverse range of experiences of LBP regarding functional impact and episode duration. The analysis is set within what we believe to be the largest trial internationally of digital self-management for LBP in primary care, with perspectives of both patient and physiotherapist participants. There are some limitations: As in the trial, there was limited ethnic diversity in our qualitative sample; consequently, apparent processes need to be confirmed in more ethnically diverse groups. Despite a purposive sampling approach, the views are of those who completed trial processes and responded to the request to be interviewed. Those who dropped out early or did not take part in the trial may have had different views and experiences.

A key test of the usefulness of the qualitatively derived theory we present is whether it can be used to improve the effectiveness of digital or hybrid digital interventions for LBP. Based on our theory, for digital self-management support focused on physical activity/exercise to have greater average impact, we need to support as many as possible to experience the positive associative process; for example, *when* they engage in physical activity or exercise, *they experience* LBP-related improvements. Positive secondary outcomes in our trial and cost-effectiveness, particularly for the unsupported intervention, suggest this was happening for some participants. However, for many, the blocks/barriers to this experience will be multifaceted and complex. Even with more sophisticated artificial intelligence to drive personalisation of activity recommendations for those with LBP, it is unlikely a digital intervention alone would be able to address and remove these barriers. Thus, future research targeting increased average effectiveness should focus on when and how to best integrate physiotherapist/clinician assessment and support. From our trial, we learnt that the model of brief standardised support calls for all was not the best use of clinician support, having little impact.^13^ Alternatively, specifically targeted, brief in-person assessment and support, focusing on barriers to effective physical activity/exercise, may be enough to remove these blocks for some patients. These barriers may be driven by physical and/or environmental factors as well as psychological processes which physiotherapists/clinicians may be able to address. Digital interventions can then be deployed to support ongoing use of physical activity to regulate LBP at minimal cost. Future research should address this. Regarding the clinical implications of this analysis, in box 1, we suggest clinical indicators that may act as barriers to physical activity and exercise-focused digital self-management for LBP. It may be useful to consider these indicators when triaging individuals for their appropriateness for such digital interventions.

Box 1 **Clinical indicators that may act as barriers to physical activity focused digital self-management for low back pain (LBP)**

1. Those with negative expectations: that is, a history of multiple unsuccessful treatments, scepticism about exercise interventions improving their LBP.
2. Those with strong beliefs about a structural cause for their LBP: (eg, slipped disc, lumbar stenosis or degenerative vertebrae).
3. Those with severe functional limitations: that is, significant physical limitations or additional health issues that hinder participation in physical activities.
4. Those with complex histories of LBP, that is, long and complex histories of LBP and previous negative healthcare experiences.
5. Those with a high need for personalisation: that is, indicates previous treatment has lacked sufficient tailoring and distrust in individualisation from digital interventions.
6. Those with psychological barriers: that is, presence of severe anxiety, depression or other psychological factors affecting engagement and motivation.

To conclude, the limited average benefit of the SupportBack intervention when added to usual primary care may have been partly driven by variable activation of an associative mechanism whereby physical activity/exercise was linked to improvements in LBP. Future research should investigate whether amendments based on these processes (eg, working to remove specific barriers) can improve the effectiveness of digital self-management interventions.

## Supplementary material

10.1136/bmjopen-2025-103428 online supplemental file 1

## Data Availability

Data are available upon reasonable request.
