# Supplementary material for: Understanding internet-supported self-management for low back pain in primary care: a qualitative process evaluation of the SupportBack 2 randomised controlled trial
Source: BMJ Open. 2025 Oct 20;15(10):e103428. doi: 10.1136/bmjopen-2025-103428 (PMC12542526; doi:10.1136/bmjopen-2025-103428)
Supplement: online supplemental file 1 [file bmjopen-15-10-s001.docx]

**Supplementary material**

**Interview topic guides for the SupportBack 2 qualitative process evaluation**

We include the topic guide for the 12-month interviews for trial participants, as this contains the same questions as three months and six months, plus some additional questions regarding longer term outcomes.

Topic guides for trial participants page 2

Topic guide for the trial physiotherapists page 11

## **Interview Schedule for participants randomised to Usual Care + SupportBack + Physiotherapist support**

**Background**

1.) Can you tell me about your experience of back pain? I am particularly interested in the events before starting the trial.

- - When did your back pain first start?
  - How did it impact on your life?

2.) I’m interested in how your back pain might have affected relationships with your friends and family. Can you tell me if and how you think it may have had an impact?

- - Are they supportive? How are they supportive?
  - If back pain has had a detrimental effect, how?

2b.) Has this had an impact on your pain? If so, how?

3.) Previous to the trial, can you tell me about any treatments/advice you have tried for your back pain?

- - Can you tell me about the most helpful treatments you have tried for your back pain?
  - Can you tell me about the least helpful treatments you have tried for your back pain?

**SupportBack Experiences**

4.) Could you tell me all about your experience of the study?

- - Can you tell me about what you expected when you first heard about SupportBack?
  - Can you tell me about how you found using SupportBack at first?
  - Can you tell me about how you found using SupportBack later on?
  - Can you tell me about what you thought was good about the intervention?
  - Can you tell be about anything you didn’t like or found difficult?

*Along with following-up where the P takes the discussion, if P mentions beliefs about movement or severity of pain, or beliefs about activity prompt for expansion*

5.) Can you tell me about your levels of activity over the course of the SupportBack programme’?

- - *If increased/same/decreased:* ‘Can you tell me more about that?’
  - Did you prefer back exercises or walking? Can you tell me more about that?

6.) I understand your experiences (and your answers to these questions) might be effected by COVID-19 and lockdown. Can you tell me if and how it has impacted you:

- In relation to your back pain
- In relation to your activity levels

7.) Can you tell me what you thought was the most useful aspect of the SupportBack website?

8.) Can you tell me how you used the SupportBack website? (If unsure, how often did they log on, how long did they say on it each times etc.)

- - *If high or low use*: ‘Can you tell me more about that?’

9.) How do you feel the treatment you received in the Support Back trial compared with other treatments you have tried for your back pain?

**Questions about telephone physio support**

*Through the general questions below, listen for and prompt expansion when a patient mentions anything related to the relationship between patient and the physiotherapist, or physiotherapist characteristics (for instance talk around “listening” “caring” ”‘felt I could tell them how I was”.*

10.) Can you tell me about your experience of telephone support from the physiotherapist?

- Can you tell me about what you thought was good about it? What did you value most about the telephone calls?
- Can you tell be about anything you didn’t like or found difficult?
- Can you tell me about anything you would change?

11.) Can you tell me about having access to the website and having the calls, together, as a package?

- Was it useful/Not useful, if so, why?

12.) How important was it to you that the person you spoke to was knowledgeable about back pain?

- How would you feel about receiving a phone call from somebody who is not a back pain expert?
- Do you think there would be benefit from receiving calls from a non-expert?

**GP experiences**

13.) Can you tell me about your experiences with your GP in relation to your back pain prior to the trial?

14.) Can you tell me about any back related experiences or interactions you have had with your GP since starting the trial?

**Impact**

15.) Can you tell me about any changes that have occurred as you’ve been taking part in the Support Back trial?

- ‘Changes in your day-to-day activities?’ [Function]
- ‘Changes in your experience of pain, or how troubled you have been by pain?’ [Pain severity, troublesomeness]
- Changes in lifestyle or habits? Might these have impacted your pain?
- Changes in the extent to which back pain interferes with work / changes to the amount of pain relief needed / changes in the need for consultations

*If patient suggests a positive impact on any of the above prompt with the following:*

15a.) Can you tell me all about how you think that might have happened?

15b.) Can you tell me what you felt was most important in bringing about this change?

16.) It’s now over 12 months since you started in the trial. Can you tell me how you have found managing your back pain as time has passed? Since the early part of the study when you first had access to the website [and physiotherapist support where applicable]?

- Prompt for ways techniques learnt from the trial may (or may not) have been integrated into everyday life.
- What have you done / are you doing for your back pain now?
  - If still doing SB exercises probe for motivations – what makes you keep doing them?
- If they describe changes probe for whether changes are in intensity / duration / frequency.

17.) If you experienced another bout of back pain now, would you manage it any differently to before the trial? (if having an episode of pain at the time of interview, ask if they are managing it differently now than they would before the trial)

18.) How do you feel about your back pain now?

- How do you feel emotionally about your back?
- How do you view/perceive your back pain now?
- Is this different to how you felt at the start of the trial?

19.) How do you see the future of your back pain?

- Is this different to how you saw the future of your back pain at the start of the trial?

**Opinions about the SupportBack Treatment**

20.) Looking back on your experiences in the trial so far, do you have any particular thoughts or feelings about the use of this treatment for back pain?

- - Thinking about the pattern of your back pain, at what point do you think the intervention would have been most helpful?
  - Do you think the intervention / techniques / exercises should be used continually, or when people have an episode of back pain?
  - When in your back pain journey do you think this intervention would have been most helpful – e.g. right at the beginning of your pain, or after a while?

## **Interview Schedule for participants randomised to Usual Care + SupportBack**

**Background**

1.) Can you tell me about your experience of back pain? I am particularly interested in the events before starting the trial.

- - When did your back pain first start?
  - How did it impact on your life?

2.) I’m interested in how your back pain might have affected relationships with your friends and family. Can you tell me if and how you think it may have had an impact?

- - Are they supportive? How are they supportive?
  - If back pain has had a detrimental effect, how?

2b.) Has this had an impact on your pain? If so, how?

3.) Previous to the trial, can you tell me about any treatments/advice you have tried for your back pain?

- - Can you tell me about the most helpful treatments you have tried for your back pain?
  - Can you tell me about the least helpful treatments you have tried for your back pain?

**SupportBack Experiences**

4.) Could you tell me all about your experience of the study?

- - Can you tell me about what you expected when you first heard about SupportBack?
  - Can you tell me about how you found using SupportBack at first?
  - Can you tell me about how you found using SupportBack later on?
  - Can you tell me about what you thought was good about the intervention?
  - Can you tell be about anything you didn’t like or found difficult?

*Along with following-up where the P takes the discussion, if P mentions beliefs about movement or severity of pain, or beliefs about activity prompt for expansion*

5.) Can you tell me about your levels of activity over the course of the SupportBack programme’?

- - *If increased/same/decreased:* ‘Can you tell me more about that?’
  - Did you prefer back exercises or walking? Can you tell me more about that?

6.) I understand your experiences (and your answers to these questions) might be effected by COVID-19 and lockdown. Can you tell me if and how it has impacted you:

- In relation to your back pain
- In relation to your activity levels

7.) Can you tell me what you thought was the most useful aspect of the SupportBack website?

8.) Can you tell me how you used the SupportBack website? (If unsure, how often did they log on, how long did they say on it each times etc.)

- - *If high or low use*: ‘Can you tell me more about that?’

9.) How do you feel the treatment you received in the Support Back trial compared with other treatments you have tried for your back pain?

**GP experiences**

13.) Can you tell me about your experiences with your GP in relation to your back pain prior to the trial?

14.) Can you tell me about any back related experiences or interactions you have had with your GP since starting the trial?

**Impact**

15.) Can you tell me about any changes that have occurred as you’ve been taking part in the Support Back trial?

- ‘Changes in your day-to-day activities?’ [Function]
- ‘Changes in your experience of pain, or how troubled you have been by pain?’ [Pain severity, troublesomeness]
- Changes in lifestyle or habits? Might these have impacted your pain?
- Changes in the extent to which back pain interferes with work / changes to the amount of pain relief needed / changes in the need for consultations

*If patient suggests a positive impact on any of the above prompt with the following:*

15a.) Can you tell me all about how you think that might have happened?

15b.) Can you tell me what you felt was most important in bringing about this change?’

16.) It’s now over 12 months since you started in the trial. Can you tell me how you have found managing your back pain as time has passed? Since the early part of the study when you first had access to the website?

- Prompt for ways techniques learnt from the trial may (or may not) have been integrated into everyday life.
- What have you done / are you doing for your back pain now?
  - If still doing SB exercises probe for motivations – what makes you keep doing them?
- If they describe changes probe for whether changes are in intensity / duration / frequency.

17.) If you experienced another bout of back pain now, would you manage it any differently to before the trial? (if having an episode of pain at the time of interview, ask if they are managing it differently now than they would before the trial)

18.) How do you feel about your back pain now?

- How do you feel emotionally about your back?
- How do you view/perceive your back pain now?
- Is this different to how you felt at the start of the trial?

19.) How do you see the future of your back pain?

- Is this different to how you saw the future of your back pain at the start of the trial?

**Opinions about the SupportBack Treatment**

20.) Looking back on your experiences in the trial so far, do you have any particular thoughts or feelings about the use of this treatment for back pain?

- - Thinking about the pattern of your back pain, at what point do you think the intervention would have been most helpful?
  - Do you think the intervention / techniques / exercises should be used continually, or when people have an episode of back pain?
  - When in your back pain journey do you think this intervention would have been most helpful – e.g. right at the beginning of your pain, or after a while?

## **Interview Schedule for participants randomised to Usual Care**

**Background**

1.) Can you tell me about your experience of back pain? I am particularly interested in the events before starting the trial.

- - When did your back pain first start?
  - How did it impact on your life?

2.) I’m interested in how your back pain might have affected relationships with your friends and family. Can you tell me if and how you think it may have had an impact?

- - Are they supportive? How are they supportive?
  - If back pain has had a detrimental effect, how?

2b.) Has this had an impact on your pain? If so, how?

3.) Previous to the trial, can you tell me about any treatments/advice you have tried for your back pain?

- - Can you tell me about the most helpful treatments you have tried for your back pain?
  - Can you tell me about the least helpful treatments you have tried for your back pain?

**SupportBack 2 Trial Experiences**

4.) Could you tell me all about your experience of the study?

- - Can you tell me about what you expected when you first heard about SupportBack?

*Along with following-up where the P takes the discussion, if P mentions beliefs about movement or severity of pain, or beliefs about activity prompt for expansion*

5.) Can you tell me about your levels of activity over the course of the SupportBack programme’?

- - *If increased/same/decreased:* ‘Can you tell me more about that?’

6.) I understand your experiences (and your answers to these questions) might be effected by COVID-19 and lockdown. Can you tell me if and how it has impacted you:

- In relation to your back pain
- In relation to your activity levels

**GP experiences**

7.) Can you tell me about your experiences with your GP in relation to your back pain prior to the trial?

8.) Can you tell me about any back related experiences or interactions you have had with your GP since starting the trial?

**Impact**

9.) Can you tell me about any changes that have occurred as you’ve been taking part in the SupportBack 2 trial?

- ‘Changes in your day-to-day activities?’ [Function]
- ‘Changes in your experience of pain, or how troubled you have been by pain?’ [Pain severity, troublesomeness]
- Changes in lifestyle or habits? Might these have impacted your pain?
- Changes in the extent to which back pain interferes with work / changes to the amount of pain relief needed / changes in the need for consultations

*If patient suggests a positive impact on any of the above prompt with the following:*

9a.) Can you tell me all about how you think that might have happened?

9b.) Can you tell me what you felt was most important in bringing about this change?’

10.) It’s now over 12 months since you started in the trial. Can you tell me how you have found managing your back pain as time has passed?

- What have you done / are you doing for your back pain now?
- If they describe changes probe for whether changes are in intensity / duration / frequency.

18.) How do you feel about your back pain now?

- How do you feel emotionally about your back?
- How do you view/perceive your back pain now?
- Is this different to how you felt at the start of the trial?

19.) How do you see the future of your back pain?

- Is this different to how you saw the future of your back pain at the start of the trial?

# **SupportBack 2 Interview Schedule for Research Physiotherapists**

## **Questions**

### Overall views of providing telephone support:

1.) Can you tell me all about your overall experience of providing telephone support in the trial?

- What went well with the telephone calls from your perspective?
- Was there anything that you found particularly challenging or difficult?
- How did you feel providing telephone support in the Support Back trial? [Probe if they are used to delivering remote consultations and how this is similar / different to the research trial]

2.) Has your attitude towards self-management strategies for people with back pain changed at all as a result of taking part in the study? [Probe: knowledge, skills, confidence]

- If so, why?

3.) Were there any practical issues that affected your ability to deliver the telephone support?

- For example, interruptions, signal, participant forgetting they were due to receive a call.

### Physio perception of patient view/experience

4) Did you get any feedback from patients about how they found receiving the telephone support? [Probe: experience, outcome, recommend to others etc]

5.) How confident were they in using this new approach (telephone and online)?

6.) Were there any patients / groups of patients for whom the intervention appeared particularly appropriate or inappropriate?

- If so, can you tell me a bit more about that, for example, for whom and why?

### Patient – Physio relationship

7.) Can you tell me about the relationships you developed with patients whilst supporting their use of the internet intervention? [Probe: opening consultations, rapport etc]

8.) How do you think the phone calls impacted participant engagement with the website?

9.) I’m interested in whether you managed to pick up on how participants were using the website. For example, before or between physio calls? Could you tell me a bit about this?

- Do you remember any examples you could share?

10.) How did having the website as a focus point affect the physio/patient relationship?

### Limitations and Future research:

11.) Was there anything at all that you would change about a) the website; b) the timing and frequency of the phone calls; c) the study? If so, what?

12.) Was there anything that you would change about your role? If so, what?

13.) Do you have any advice / suggestions for the research team?

14.) Do you have any other thoughts you’d like to share?
