# Supplementary material for: Understanding internet-supported self-management for low back pain in primary care: a qualitative process evaluation of the SupportBack 2 randomised controlled trial
Source: BMJ Open. 2025 Oct 20;15(10):e103428. doi: 10.1136/bmjopen-2025-103428 (PMC12542526; doi:10.1136/bmjopen-2025-103428)
Supplement: Supplementary file 2 [file bmjopen-2025-103428.reviewer_comments.pdf]

PEER REVIEW HISTORY

BMJ Open publishes all reviews undertaken for accepted manuscripts. Reviewers are asked to complete a checklist review form and are provided with free text boxes to elaborate on their assessment. These free text comments are reproduced below.

ARTICLE DETAILS

Title (Provisional)

Understanding internet supported self-management for low back pain in primary care: A qualitative process evaluation of the SupportBack 2 randomised controlled trial.

Authors

Geraghty, Adam W A; hughes, Stephanie; Roberts, Lisa; Hill, Jonathan C; Foster, Nadine E.; Hay, Elaine; Mansell, Gemma; White, Malcolm; Davies, Firoza; Steele, Mary; Little, Paul; Yardley, Lucy

---

VERSION 1 - REVIEW

**Reviewer**	**1**
**Name**	**Kowalski, Katie**
**Affiliation**	**Western University, School of Physical Therapy**
**Date**	**19-Jun-2025**
**COI**	**None**

This qualitative study was a process evaluation of the SupportBack 2 trial, which was designed to determine the clinical and cost-effectiveness of an internet intervention in supporting self-management of LBP versus usual primary care. Trial patient and physiotherapist participants took part in semi-structured interviews to understand the processes and potential mechanisms underlying the impact of the intervention, specifically focusing on context-mechanism-outcome relationships. Process evaluations are an important part of testing complex interventions, and this study adds value to the literature by highlighting key clinical features where barriers may exist limiting effectiveness of internet-based interventions for self-management of LBP and identifies new research questions to understand further how to best manage LBP, a leading cause of disability internationally. The study is clear and well-written. I have no major concerns, but a few minor points to address, which are detailed below.

Introduction

• Very clear, flows well and the setup is logical.

• A key sentence in setting up the gap for this study is the last sentence of paragraph 3 (starts on page 4, line 57). This sentence is 5 lines long and complex, making it difficult to read. It can be improved to aid readability.

• Page 5, Line 17: define MSK before abbreviating

• Can the authors justify the process evaluation focus on behavioural?

Methods

• Please add reporting guidelines to design paragraph

• Trial participants sampling and recruitment paragraph (Page 6, Line 40):

o Please elaborate on how the various relevant items that contribute to information power (e.g., aim, sample specificity) were considered to inform sample size

o Describes recruitment of trial participants at each time point, but the reader has not yet been oriented to what the time points are with regards to process evaluation. This is described in subsequent paragraphs, but it may be useful to provide a brief overview in the first design paragraph.

• Physiotherapist sampling and recruitment para (Page 6, line 53): Can you please clarify if this was total population sampling?

• Authors could consider adding a paragraph bringing together all techniques used to enhance trustworthiness, to be explicit rather than having the reader infer

Results

• Can you please clarify the percent of trial participants agreed to be interviewed? For example, I'm not clear if the 92 trial participants invited was the total population of the trial, or a subset.

• Page 17, lines 34-46: There are many uses of the word 'support', e.g., PTs supported by study team, PTs delivering support. These many uses makes explanatory theme 3 (validating support) difficult to interpret who is providing support for what, until the very end of the paragraph on line 17. Can the authors please enhance clarity?

• Figure 1 is great. Just a couple minor points:

o Spelling error (SupportBac)

o Consider if it is useful to label the sections of context, mechanisms, outcomes

o I'm unsure about the arrow with the big 'X' going through it. Is it needed because the grey box already separates the effective self-management pathway, suggesting cannot move from the lower pathways to the upper pathway? If it represents something else, then maybe a brief description of the arrow + 'X' would be useful.

Discussion

• Discussion is well-written and easy to follow.

• Is there also opportunity to identify the homogenous subgroup that would benefit from a digital intervention alone or with minimal support from a healthcare professional?

• Box 1 is a useful clinical summary. Is there a reason for focusing on barriers? Are there positive clinical indicators that could be highlighted, to balance the presentation?

**Reviewer**	**2**
**Name**	**Zacharoff, Kevin**
**Affiliation**	**Renaissance School of Medicine at Stony Brook University**
**Date**	**20-Jul-2025**
**COI**	**None**

While the study sample is relatively small, this manuscript is thoughtful, intelligent, pertinent, and very well written. It seems unquestionable that interventions such as the one described will likely be a part of the future for the treatment of patients with refractory low back pain. If there is any constructive comment to be made, it would be to outline the intervention to the same degree that the surveys were. Excellent job!

VERSION 1 - AUTHOR RESPONSE

**Reviewer 1**
This qualitative study was a process evaluation of the SupportBack 2 trial, which was designed to determine the clinical and cost-e>ectiveness of an internet intervention in supporting self-management of LBP versus usual primary care. Trial patient and physiotherapist participants took part in semi-structured interviews to understand the processes and potential mechanisms underlying the impact of the intervention, specifically focusing on context-mechanism-outcome relationships. Process evaluations are an important part of testing complex interventions, and this study adds value to the literature by highlighting key clinical features where barriers may exist limiting e>ectiveness of internet-based interventions for self-management of LBP and identifies new research questions to understand further how to best manage LBP, a leading cause of disability internationally. The study is clear and well-written. I have no major concerns, but a few minor points to address, which are detailed below.
We thank the reviewer for their positive comments and useful minor points that we have addressed below.
Introduction<br>Very clear, flows well and the setup is logical.
Thank you.
A key sentence in setting up the gap for this study is the last sentence of paragraph 3 (starts on page 4, line 57). This sentence is 5 lines long and complex, making it di>icult to read. It can be improved to aid readability.

This is a good point. We have now split the original overly long sentence. This can be found on page 4, paragraph 3 of the revised manuscript. We have also copied below and highlighted the changes for easy of review:<br><br>"In both trials there were indications of some benefit i.e. small but statistically significant improvements in LBP-related disability at 3 months in the selfBACK trial [12] and cost-effectiveness in the SupportBack trial (particularly for the unsupported intervention) [13]. However, the average between group effect of these digital interventions on LPB-related disability compared to usual care was very modest."
Page 5, Line 17: define MSK before abbreviating
This has now been corrected.
Can the authors justify the process evaluation focus on behavioural?
Whilst SupportBack is inherently a behavioural intervention, we agree that the process evaluation also included other aspects such as cognitive and a>ective processes. Thus, we have now removed 'behavioural' from the sentence on the second paragraph on page 5. It now reads:

"We aimed to understand the processes underlying outcomes, drawing from the experiences of participants and physiotherapists involved in the trial."
Methods<br>• Please add reporting guidelines to design paragraph
We have now added this to the design paragraph on page 5:<br><br>We present a nested qualitative process study as part of the SupportBack 2 RCT (ISRCTN14736486) [13]. We followed the Standards for Reporting Qualitative Research (SRQR) [16].
• Trial participants sampling and recruitment paragraph (Page 6, Line 40):<br>o Please elaborate on how the various relevant items that contribute to information power (e.g., aim, sample specificity) were considered to inform sample size
We thank the reviewer for this point. We have now expanded on how items from the information power model were applied in our study, in the last paragraph on page 6:<br><br>"The concept of information power [20] was then used to inform when recruitment at each time point was deemed sufficient to meet the aims of the process evaluation. According to the information power model, sufficiency in a qualitative study with a 'narrow' study aim and a high level of sample specificity (i.e. a process evaluation within an RCT) is likely to be reached with relatively modest numbers. Within this context, 'quality of dialogue' was a key item used in deciding when to stop recruiting.

o Describes recruitment of trial participants at each time point, but the reader has not yet been oriented to what the time points are with regards to process evaluation. This is described in subsequent paragraphs, but it may be useful to provide a brief overview in the first design paragraph.
The paragraph describing the timepoints for interviews in the process evaluation has now been moved up, as suggested, so it appears in the first design section (in paragraph 3, on page 6). It still appears after the description of the RCT and intervention, as we feel it is important for readers to understand the focus of the qualitative process evaluation, before reading about the evaluation interview time points.
• Physiotherapist sampling and recruitment para (Page 6, line 53): Can you please clarify if this was total population sampling?
We have now clarified that we were looking to use total population sampling with the trial physiotherapists, in paragraph 2 on page 7:<br><br>"Physiotherapists: Twelve physiotherapists were involved in providing telephone support over the course of the trial, and all physiotherapists who were contactable were invited for an interview after the intervention period for trial participants (attempting total population sampling)."
• Authors could consider adding a paragraph bringing together all techniques used to enhance trustworthiness, to be explicit rather than having the reader infer
We have now added a brief paragraph that brings together techniques used to enhance trustworthiness, on page 9, paragraph 2:

"*Trustworthiness*<br>Many of the above approaches were used to enhance the trustworthiness [27] of the analysis: Initial double coding; sharing of detailed codebooks across the team including public contributors; triangulation of the on-going analysis with public contributors and multidisciplinary co-authors; sequential analysis of trial participant and physiotherapist participant data, and a reflexive approach throughout."
Results<br>• Can you please clarify the percent of trial participants agreed to be interviewed? For example, I'm not clear if the 92 trial participants invited was the total population of the trial, or a subset.
Thank you for this point. We have now clarified that the 92 trial participants were invited from the total trial population of 825 (based on our purposive sampling frame).<br><br>"Ninety-two trial participants were invited to interview (from the total RCT sample, n = 825) across three time points within the trial (three months, six months and 12 months post randomisation). Forty-six participants agreed to interview (three months n = 15, six months n = 14, 12 months n=17)."<br><br>Whilst we list the total trial number as a point of clarification, we would prefer not to list the percentage of trial participants that agreed to be interviewed. Although this can be calculated from figures provided (5.5%), directly stating it risks imposing a quantitative framing on this qualitative study, where purposive sampling and data adequacy guide rigour rather than representativeness/ % of RCT sample.

• Page 17, lines 34-46: There are many uses of the word ‘support’, e.g., PTs supported by study team, PTs delivering support. These many uses makes explanatory theme 3 (validating support) di>icult to interpret who is providing support for what, until the very end of the paragraph on line 17. Can the authors please enhance clarity?

This is a very good point. We thank the reviewer for noting it. We feel it is the “supported by the study team” that makes paragraph confusing. We have now replaced supported, with “well-guided”. The term support is now only used with reference to the physiotherapists providing support to the patient participants in the trial.

“There were some minor implementation issues such as difficulty in contacting some of the participants, or participants favouring call times that were difficult for the physiotherapists. The physiotherapists reported feeling well-guided by the study team and equipped to deliver the support following their training as part of the trial.”

• Figure 1 is great. Just a couple minor points:
o Spelling error (SupportBac)

We could not find this spelling error, but have now uploaded the figure as a PDF to ensure formatting issues do not a>ect spelling within the boxes.

o Consider if it is useful to label the sections of context, mechanisms, outcomes

We have considered this suggestion but decided not to add these labels to the figure. The figure is a general overview, and we would prefer to keep those terms for the specific CMO configurations listed in the main results section of the paper.

o I’m unsure about the arrow with the big ‘X’ going through it. Is it needed because the grey box already separates the e>ective self-management pathway, suggesting cannot move from the lower pathways to the upper pathway? If it represents something else, then maybe a brief description of the arrow + ‘X’ would be useful.

We are grateful to the reviewer for this suggestion. We agree that removing the arrow with the grey X going through it simplifies the figure. It has been removed.

Discussion
• Discussion is well-written and easy to follow.

Thank you.

• Is there also opportunity to identify the homogenous subgroup that would benefit from a digital intervention alone or with minimal support from a healthcare professional?

We thank the reviewer for this interesting suggestion. We are not sure we can address that based on our qualitative study. Thus, we have not added anything to the paper.<br><br>However, based on the theory proposed, it is unlikely that the subgroup who benefit from a digital intervention alone would be homogenous in terms of characteristics. It may be that the group who benefit from unguided interventions, are those who can apply them such that they start to perceive benefits. These perceived benefits/improvements then reinforce the behaviour. Further determining *who* is able to do this warrants future research.
• Box 1 is a useful clinical summary. Is there a reason for focusing on barriers? Are there positive clinical indicators that could be highlighted, to balance the presentation?
When considering direct clinical applications from our analysis, the barriers that can be derived are more concrete, thus supporting direct clinical application. Positive clinical indicators that could be easily applied are the implied reverse of the barriers listed (e.g. positive expectations, absences of belief re structural cause).
**Reviewer 2**
While the study sample is relatively small, this manuscript is thoughtful, intelligent, pertinent, and very well written. It seems unquestionable that interventions such as the one described will likely be a part of the future for the treatment of patients with refractory low back pain. If there is any constructive comment to be made, it would be to outline the intervention to the same degree that the surveys were. Excellent job!
We thank this reviewer for these very positive comments. To keep the paper a manageable length, we have added some further detail on behavioural theories used in the intervention and a further reference to where readers can find more detailed descriptions of the intervention in the last paragraph on page 5:

"The internet intervention 'SupportBack' has been described in full elsewhere [10, 17]. Briefly, the aim of the SupportBack intervention was to support self-management of LBP through general physical activity with a specific focus on walking and back exercises. The intervention was based primary around self-efficacy and self-regulatory theory [18, 19]. Cognitive reassurance, graded goal setting, self-monitoring and tailored feedback were used to support increases in walking or back exercises (or both)"

VERSION 2 - REVIEW

**Reviewer**	**1**
**Name**	**Kowalski, Katie**
**Affiliation**	**Western University, School of Physical Therapy**
**Date**	**21-Sep-2025**
**COI**	

Thank you for addressing my points / questions. The authors have a well-written and important manuscript. I look forwards to seeing it in the published version.
